# A critical role of autophagy in regulating the mesenchymal transition of ductular cells in liver cirrhosis

**DOI:** 10.1038/s41598-019-46764-x

**Published:** 2019-07-23

**Authors:** Tzu-Min Hung, Yu-Jen Huang, Yu-Chun Lin, Yu-Hsuan Chen, Yao-Ming Wu, Po-Huang Lee

**Affiliations:** 1 Department of Surgery, National Taiwan University Hospital and National Taiwan University College of Medicine, Taipei, Taiwan; 2 Department of Medical Research, E-DA Hospital, Kaohsiung, Taiwan; 3 Department of Surgery, E-DA Hospital, Kaohsiung, Taiwan; 4 Department of Internal Medicine, National Taiwan University Hospital and National Taiwan University College of Medicine, Taipei, Taiwan

**Keywords:** Autophagy, Mechanisms of disease

## Abstract

Our previous studies have shown that autophagy mediates the link between ductular reaction (DR) and liver cirrhosis. Whether the subsequent fibrogenic response is regulated by increased autophagy in DR remains unclear. Here, using both human liver specimens and a rat model of liver cirrhosis induced by 2-acetylaminofluorene (AAF) and carbon tetrachloride (CCL4), we explored the involvement of autophagy in regulating mesenchymal transition of ductular cells. Ductular cells from AAF/CCL4 livers exhibited increased autophagy compared to those of normal livers. These cells showed morphological and functional characteristics of mesenchymal cells. Blocking autophagy using bafilomycin A1 or siRNA targeting ATG7 reduced the expression of mesenchymal markers in these ductular cells from AAF/CCL4 livers, indicating a role for autophagy in regulating the mesenchymal phenotype of ductular cells. Furthermore, we show that the mesenchymal transition in DR requires the activation of transforming growth factor-β (TGF-β) signaling in an autophagy-dependent manner. Importantly, in cirrhotic human livers, ductular cells that are positive for LC3B also showed increased expression of TGF-β and fibroblast-specific protein-1. Our data suggest activation of autophagy in ductular cells, and also demonstrate that it is required for the mesenchymal transition during the DR, processes that are critically involved in the pathogenesis of cirrhosis.

## Introduction

Ductular reaction (DR) is a common response to injury observed in a variety of liver diseases^1^. It refers to the appearance of reactive ductular cells, which are a population of cholangiocyte-like epithelial cells that can orchestrate a complex mixture of extracellular matrix, mesenchymal cells and inflammatory infiltrate^1,2^. Accumulating evidence indicates that DR correlates closely with the severity of fibrosis in several human liver diseases^3–5^; but relatively little is known about its underlying cellular and molecular mechanisms. In addition to production of factors that recruit mesenchymal elements, transitions from ductular cells to myofibroblast through the process of epithelial-mesenchymal transition (EMT) has been proposed as a mechanism by which DR drives liver fibrosis^6,7^.

Svegliati-Baroni *et al*. demonstrated that CK7-positive cells from hepatitis C virus-positive liver show the nuclear expression of Snail, downregulation of E-cadherin and expression of fibroblast-specific protein-1 (FSP-1), providing evidence for an active EMT process in these ductular cells^8^. Rygiel *et al*. also documented the coexpression of epithelial and mesenchymal markers in the DR in human tissues and the mobilization of epithelial cells that normally reside in the ductular structure^9^. Moreover, Rygiel *et al*. found increased TGF-β mRNA expression and coexpression of FSP-1 and phosphorylated forms of Smad2/3 in the nucleus of ductular cells, demonstrating that EMT is driven by TGF-β signaling^9^.

Autophagy, a complex regulatory pathway in liver fibrosis^10^, exerts its profibrogenic effects by contributing to hepatic stellate cell activation^11,12^ and antifibrogenic effects via indirect hepatoprotective^13^ and anti-inflammatory activity^14^. Our previous studies have shown that increased expression of autophagy markers is associated with DR during the development of cirrhosis^15^, implicating autophagy as a pathological driver of liver fibrosis in the context of ductular cells. Recent work has presented evidence that starvation-induced autophagy can induce the expression of EMT markers and the invasion of hepatic carcinoma cells in a TGF-β/Smad3 signaling-dependent manner^16^. These results prompted us to study whether autophagy activation precedes and leads to EMT of ductular cells in liver cirrhosis. We used a model of cirrhosis in the rat, produced by 2-acetylaminofluorene (AAF) and carbon tetrachloride (CCL4). In this model, administration of AAF before and during CCL4 treatment blocks the proliferative hepatocyte response to CCL4-induced necrosis and leads to the emergence of an alternative regenerative pathway characterized by the periportal accumulation of ductular cells, similar to what is commonly reported in human cirrhosis^17^. Ductular cell isolation was based on positive selection approach for epithelial cell adhesion molecule (EpCAM), which is a well-established surface marker for DR. In this study, we demonstrate increased autophagy in the ductular cells isolated from AAF/CCL4 livers, and that autophagy is required for mesenchymal transition in ductular cells of AAF/CCL4 livers and in cirrhotic human livers.

## Results

### Human cirrhotic livers show increased autophagy in the ductular reaction but not in hepatocytes

**Figure 1:**
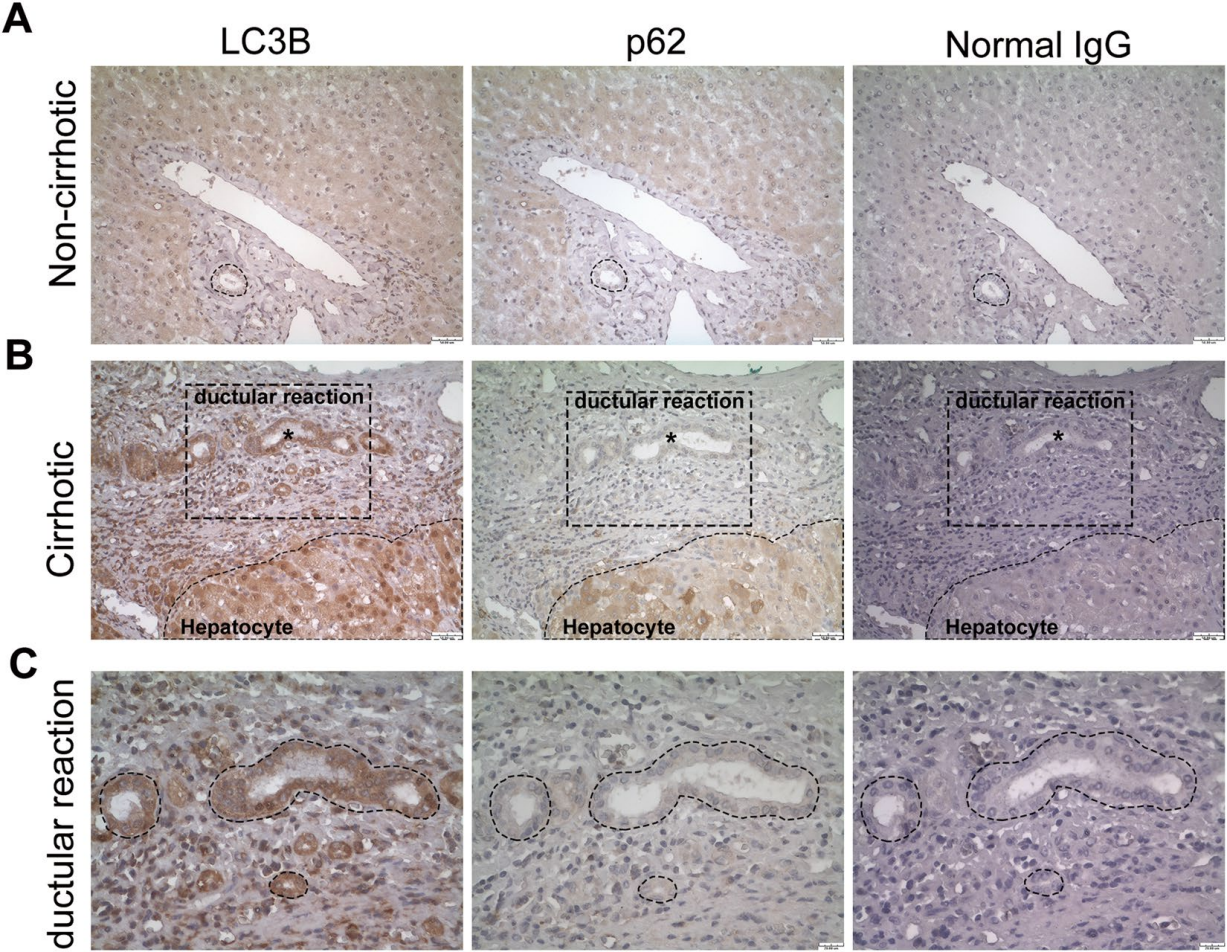
Immunohistochemical detection of autophagy markers in liver tissues from patients with cirrhosis. (**A**,**B**) Paraffin-embedded three consecutive liver sections were stained with antibodies against LC3B, p62 or normal IgG. Ductular reaction in cirrhotic liver (**B**) showed strong expression of LC3B but were negative for p62, whereas hepatocytes in cirrhotic liver were positive for both LC3B and p62. Rabbit normal IgG was used as a negative control counterstain. Asterisks indicate the ductular reaction in cirrhotic livers. Scale bar, 50 μm. (**C**) Higher magnifications of the ductular reaction in corresponding cirrhotic liver sections are enlarged micrographs from the respective boxed area. The dotted lines indicated the same region among three consecutive sections. Scale bar, 20 μm.

Our previous studies demonstrated that increased autophagy markers are associated with DR^15^. To further support this finding, we performed immunohistochemistry for p62/SQSTM1 (hereafter referred to as p62), which is an adaptor molecule that is selectively degraded via autophagy, and LC3B, which is a marker of autophagosomes, on human cirrhotic liver samples. As shown in Fig. 1A, noncirrhotic liver exhibited only weak to moderate staining of LC3B and p62. In cirrhotic liver sections, distinct LC3B and p62 staining was observed in DR and parenchymal hepatocytes (Fig. 1B and Supplementary Fig. S1). The DR in cirrhotic livers exhibited higher expression of LC3B but not p62, indicating induction of autophagy. In contrast, both LC3B and p62 were substantially increased in hepatocytes in cirrhotic livers, suggesting defective hepatic autophagy.

### Isolation and characterization of rat primary ductular cells

**Figure 2:**
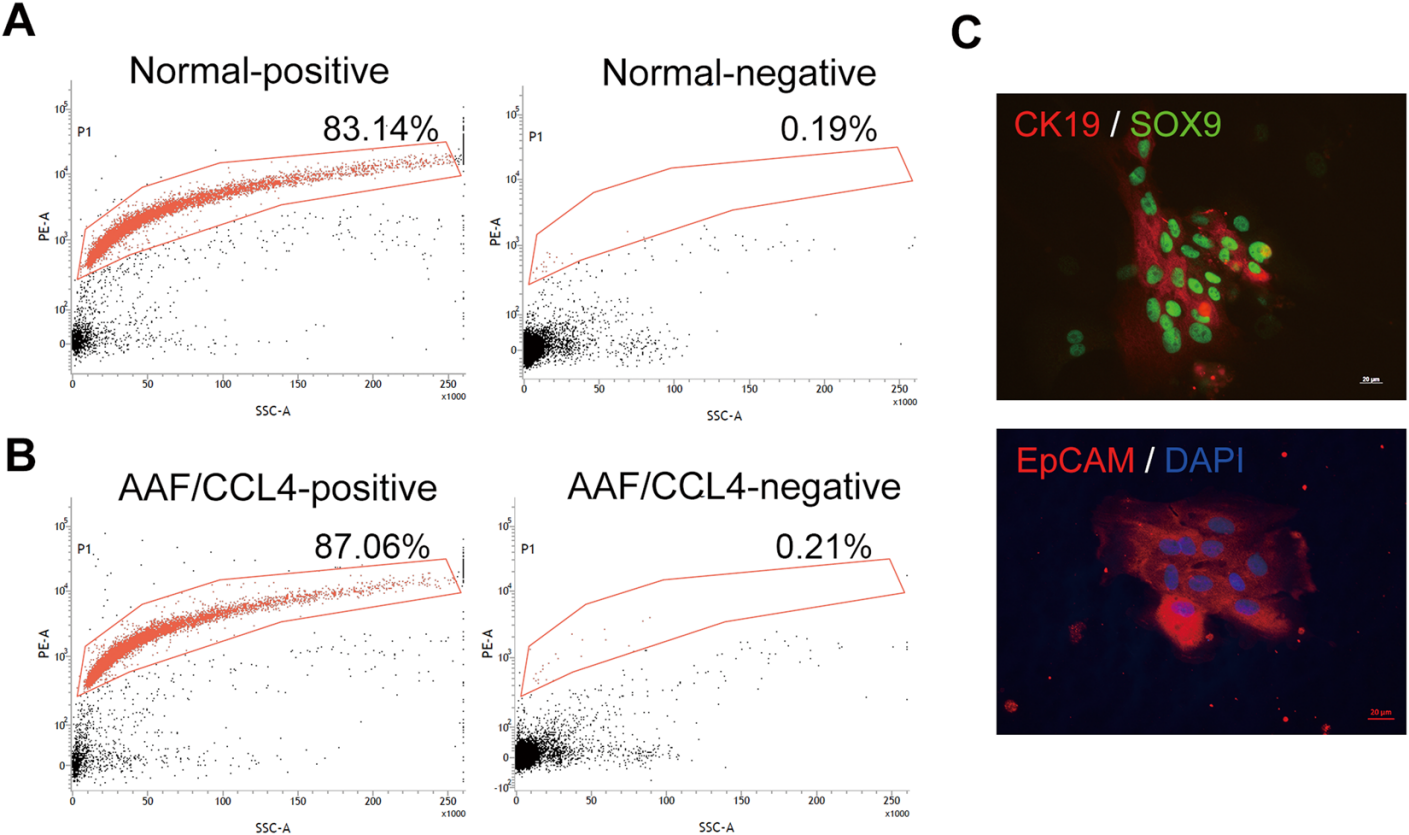
Characterization of ductular cells from AAF/CCL4-induced cirrhosis rat model. (**A**,**B**) Flow cytometry analysis of freshly isolated ductular cells from normal (**A**) and AAF/CCL4 (**B**) livers. The y-axis indicates EpCAM expression (PE fluorescence) and the x-axis represents side scatter (SSC). The positive and negative fractions of each liver were collected as described in the Materials and Methods. Percentage of cells expressing EpCAM is shown. (**C**) Identity of EpCAM-expressing cells in the positive fraction was further confirmed by staining for SOX9 and CK19.

To avoid confusion arises from the simultaneous existence of increased autophagy in DR and impaired autophagy in hepatocyte in cirrhotic livers, we used in *vitro* model to investigate the effect of autophagy on specific ductular cells of interest. Following methods that have been described previously for the isolation of cholangiocytes from patients with cirrhotic livers^18^, we successfully purified ductular cells from a rat model of AAF/CCL4-induced liver cirrhosis using EpCAM-positive selection. The isolated immunopositive fraction typically contained 70–90% EpCAM-expressing cells, whereas the negative fraction was almost entirely depleted of EpCAM-expressing cells. Representative fractionations of a normal liver and an AAF/CCL4 liver are depicted in Fig. 2A,B. The phenotype of cholangiocyte was confirmed by expression of CK19 and SOX9 in EpCAM-expressing cells (Fig. 2C).

### Autophagy is increased in ductular cells from AAF/CCL4 livers

**Figure 3:**
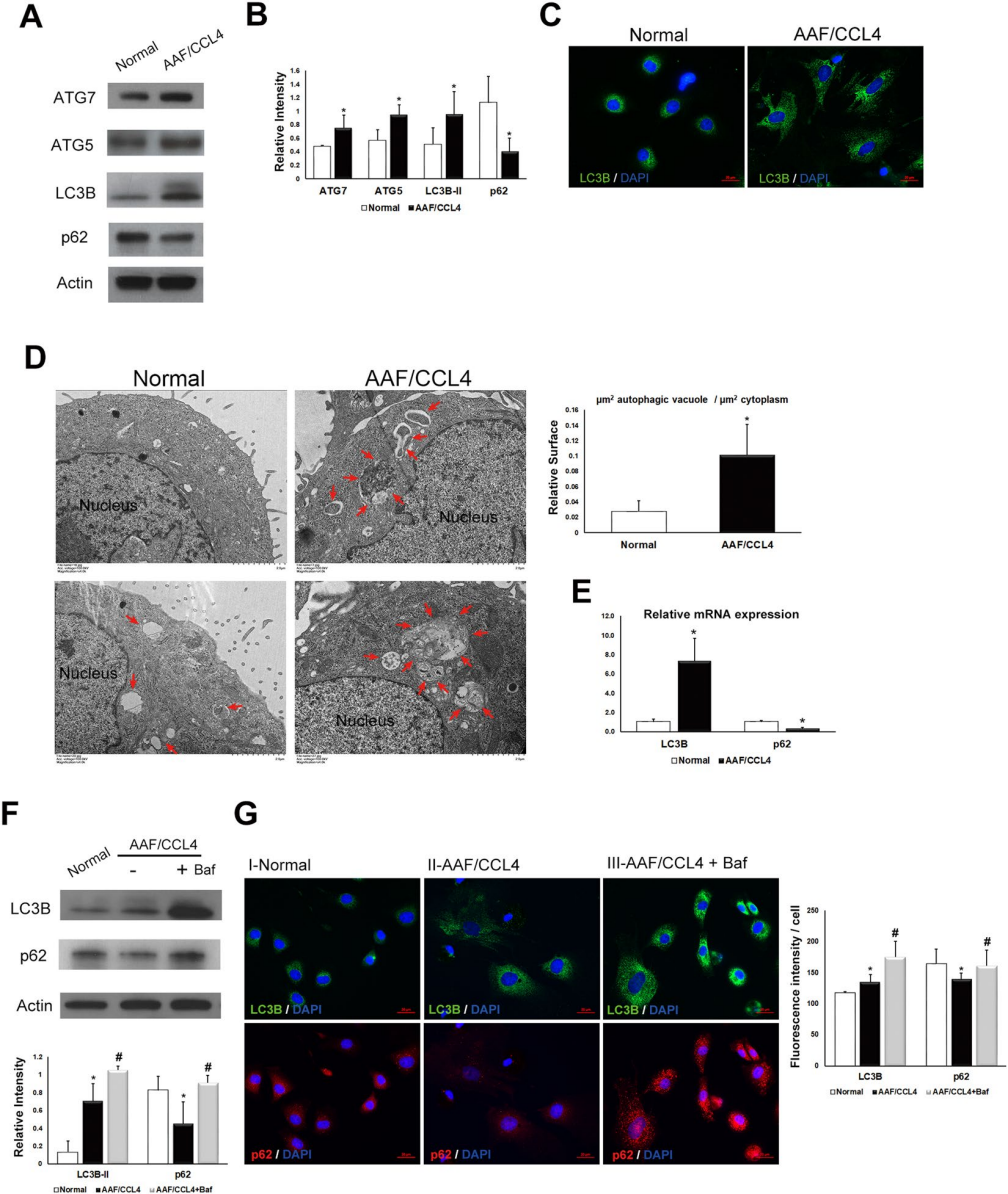
Autophagy is increased in ductular cells from AAF/CCL4 livers. (**A**) Immunoblots and (**B**) quantification depicting expression of ATG7, ATG5, LC3B-II and p62 in ductular cells from normal and AAF/CCL4 livers. β-actin served as a loading control. Full-length blots are presented in Supplementary Fig. S4. (**C**) The presence of LC3B punctae was analyzed using immunofluorescence. (**D**) Transmission electron microscopy illustrating a larger number and size of autophagic vacuoles in ductular cells from AAF/CCL4 livers than in those from normal livers. Arrows indicate autophagic vacuoles. (Right) Quantification data are expressed in relative surface of autophagic vacuole to cytoplasm. (**E**) Quantitative PCR analysis of mRNA for LC3B and p62 in ductular cells from normal and AAF/CCL4 livers. Data were normalized by the amount of β-actin mRNA, expressed relative to the corresponding value for normal cells. (**F**,**G**) Autophagic flux assay of ductular cells from normal and AAF/CCL4 livers. Immunoblots (**F**) and immunofluorescence images (**G**) depicting expression of LC3B and p62 in ductular cell. Ductular cells from AAF/CCL4 liver were treated with bafilomycin A1 (Baf, 100 nM) for 3 hours further increased the levels of both LC3B and p62, indicating increased autophagy. Full-length blots are presented in Supplementary Fig. S5. The graph underneath the blot shows the relative intensities of the LC3B-II and p62 bands normalized to β-actin from three independent experiments (mean ± SD). Quantification of immunofluorescence images was performed using Zen-Pro software (Zeiss) and normalized to the number of nuclei. Statistical analysis was analyzed by Student’s unpaired t-test. *P < 0.05 compared with normal group; ^#^*P* < 0.05 compared with AAF/CCL4 groups.

Several assays were performed to validate the role of autophagy in ductular cells from AAF/CCL4 livers. First, Western blot analysis was performed to examine autophagy-related proteins. As shown in Fig. 3A, the levels of ATG7, ATG5, and LC3B-II were significantly increased in ductular cells from AAF/CCL4 livers compared to those from normal livers. Furthermore, AAF/CCL4 ductular cells had low p62 expression than in normal ductular cells (Fig. 3A,B). Immunofluorescence analysis showed a marked increase in punctate LC3B staining in ductular cells from AAF/CCL4 livers compared with normal livers (Fig. 3C). Transmission electron microscopy was used to detect autophagy features. Ultrastructurally, numerous autophagic vacuoles were observed in ductular cells from AAF/CCL4 livers, whereas little vacuole formation was observed in normal cells (Fig. 3D). To examine whether autophagy is regulated at the transcription levels, LC3B and p62 mRNA levels were also assessed. Consistent with the protein expression data, an increase in LC3B levels and a decrease in p62 levels was observed in ductular cells from AAF/CCL4 cells than in normal cells (Fig. 3E).

Bafilomycin A1 (Baf), an inhibitor of both lysosome acidification and autophagosome-lysosome fusion, was used to further confirm that autophagic flux is increased^19^. Expression of LC3B-II in ductular cells from AAF/CCL4 livers was notably higher than that in normal cells and was further increased in the presence of Baf (Fig. 3F). While expression of p62 was reduced in AAF/CCL4 cells compared to normal ductular cells, accumulation of p62 was noted in the presence of Baf in AAF/CCL4 cells (Fig. 3F), indicating induction of autophagy. The induction of autophagy in AAF/CCL4 cells was further confirmed by immunofluorescence. A significant increase in the number of LC3B-positive punctae coupled to a decrease in the number of p62-positive puntae was observed in ductular cells from AAF/CCL4 livers than in those from normal livers (Fig. 3G). Whereas the numbers of LC3B- and p62-positive puncta were both significantly higher in AAF/CCL4 cells treated with Baf further indicated increased autophagy (Fig. 3G). Taken together, the results provide lines of evidence that increased autophagy is associated with DR during the development of cirrhosis.

### Ductular cells from AAF/CCL4 livers undergo mesenchymal transition

**Figure 4:**
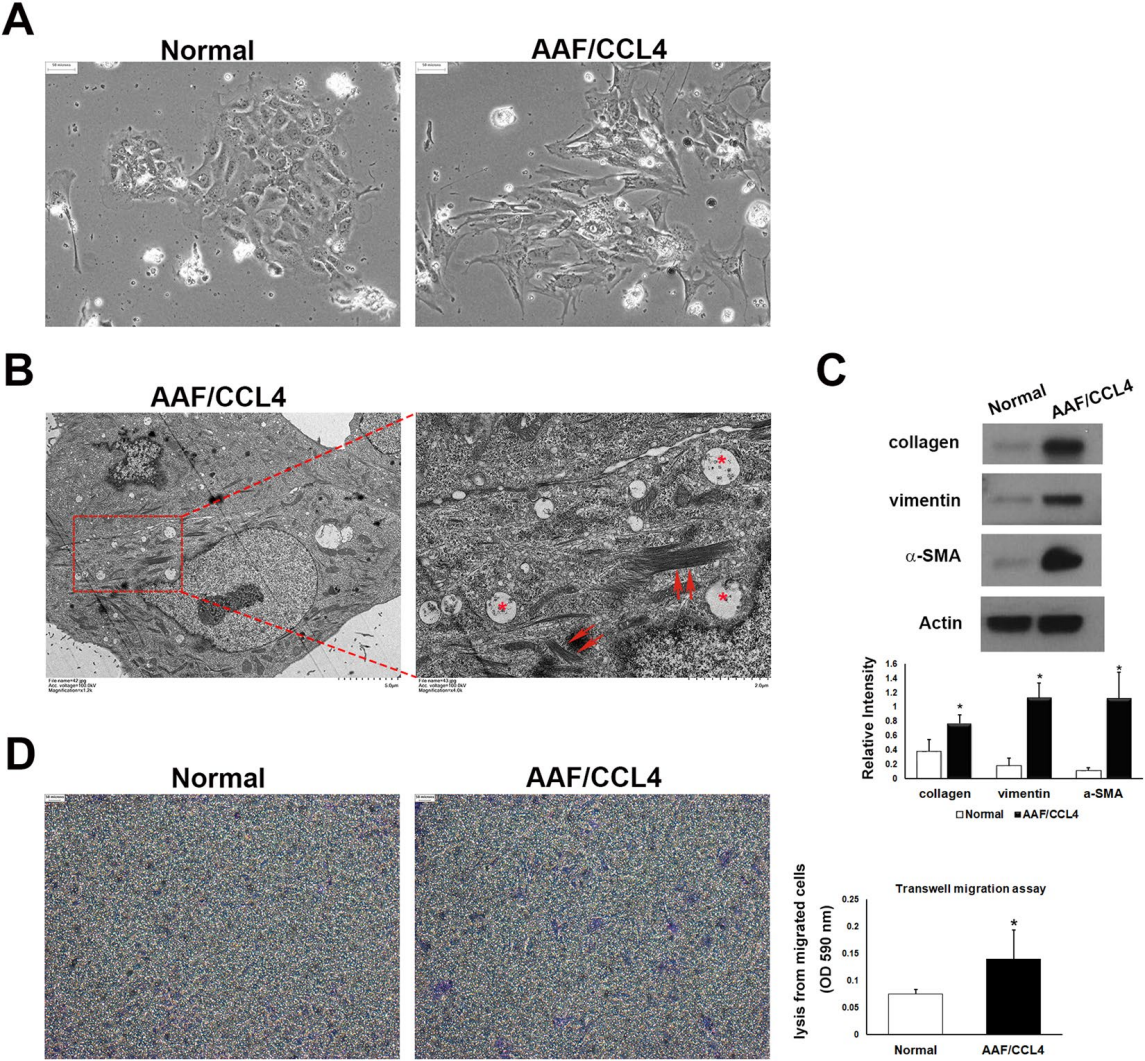
Ductular cells from AAF/CCL4 livers undergo mesenchymal transition. (**A**) Phase-contrast micrographs of ductular cells isolated from normal and AAF/CCL4 livers on day 2. Normal cells form colonies with epithelial morphology. AAF/CCL4 cells have a spindle-like morphology. (**B**) Ultrastructural analysis of ductular cells from AAF/CCL4 livers revealing accumulation of microfilaments (red arrows) and vesicles (asterisks) in the cytoplasm. The magnified image is of the area indicated by the square. (**C**) Immunoblot depicting expression of mesenchymal markers in ductular cells from normal and AAF/CCL4 livers. Full-length blots are presented in Supplementary Fig. S6. Bar graph shows the relative intensities of the type I collagen, vimentin and α-SMA bands normalized to β-actin from three independent experiments (mean ± SD) (**D**) Transwell migration assay of ductular cells isolated from normal and AAF/CCL4 livers. A statistical analysis of relative migration rate was shown on the right. Statistical analysis was analyzed by Student’s unpaired t-test. *P < 0.05 compared with normal group.

Compared with ductular cells from normal livers, those from AAF/CCL4-injured livers exhibited multiple distinguishing features, including enlarged cell size, spindle-shaped (“fibroblast-like”) appearance and loss of cell-cell adhesion. (Fig. 4A). Interestingly, ultrastructural examination demonstrated the presence of a filamentous structure that are found dispersed throughout the AAF/CCL4 cells (Fig. 4B). Following observation of features displayed by mesenchymal cells, we next assessed the changes in the expression of mesenchymal markers in AAF/CCL4 cells. Western blot analysis showed that collagen, vimentin and alpha-smooth muscle actin (α-SMA) were significantly increased in ductular cells isolated from AAF/CCL4 livers when compared with those from normal livers (Fig. 4C). Furthermore, Transwell migration assay revealed increased migration of AAF/CCL4 cells compared to normal cells at 18 hours (Fig. 4D). Collectively, ductular cells from AAF/CCL4 livers expressed a mesenchymal phenotype, which includes changes in cell morphology, expression of mesenchymal markers, and enhanced migratory capacity. These observations support the notion that ductular cells may acquire mesenchymal features during progressive liver fibrosis^20^.

### Inhibition of autophagy reduces the mesenchymal phenotype of ductular cells from AAF/CCL4 livers

**Figure 5:**
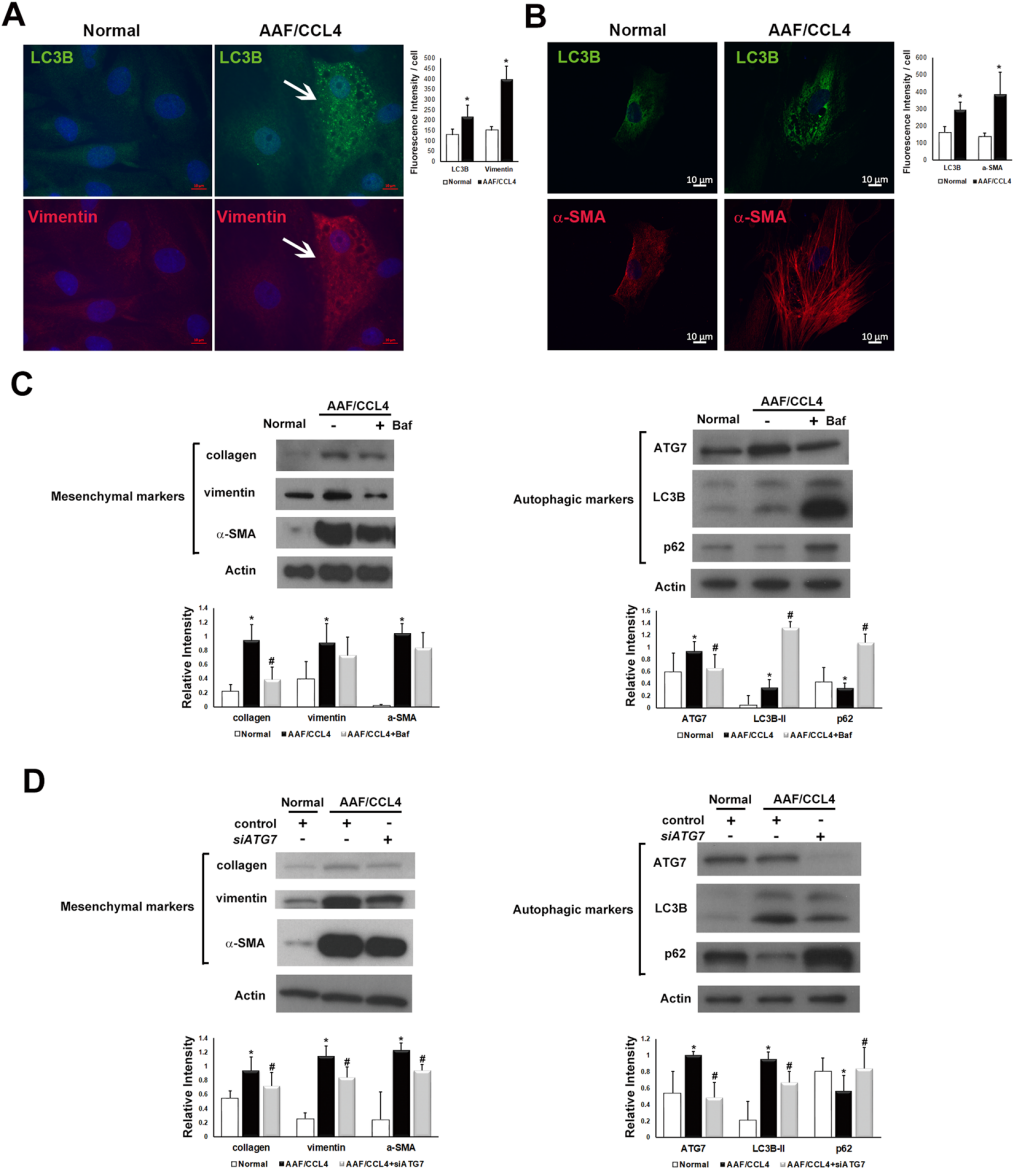
Effect of autophagy on the mesenchymal phenotype of ductular cells from AAF/CCL4 livers. (**A**,**B**) Immunofluorescence images of isolated ductular cells demonstrating LC3B was associated with the mesenchymal markers vimentin (**A**) and α-SMA (**B**). (Right) Quantification of images was performed using Zen-Pro software (Zeiss) and normalized to the number of nuclei. (**C**) Ductular cells were cultured for 3 days and then treated with or without bafilomycin A1 (Baf, 100 nM) for another 24 hours. Representative Western blot analysis confirmed the increased expression of mesenchymal markers in AAF/CCL4 cells compared to normal cells. Treatment of AAF/CCL4 cells with Baf suppressed the expression of mesenchymal markers. Full-length blots are presented in Supplementary Fig. S7. Quantitative analysis of mesenchymal markers (left panel), and autophagic markers (right panel) was shown below. (**D**) Inhibition of autophagy by *ATG7* small interfering RNA (si*ATG7*) reduced the protein levels of mesenchymal markers. Full-length blots are presented in Supplementary Fig. S8. Quantitative analysis of mesenchymal markers (left panel), and autophagic markers (right panel) was shown below. Statistical analysis was analyzed by Student’s unpaired t-test. *P < 0.05 compared with normal group; ^s#^*P* < 0.05 compared with AAF/CCL4 groups.

We then tested whether modulating autophagy alters the phenotype of ductular cells using the pharmacological inhibitor Baf or the genetic inhibitor siRNA targeting ATG7. Dual immunofluorescence revealed that expression of the autophagy marker LC3B was associated with expression of the mesenchymal markers vimentin and α-SMA in ductular cells isolated from AAF/CCL4 livers (Fig. 5A,B).

To functionally connect autophagy to mesenchymal features, we investigated the effect of Baf, which is a late-stage autophagy inhibitor, on mesenchymal phenotypes of ductular cells from AAF/CCL4 livers. As shown in Fig. 5C, Baf induced a significant increase in the expression of both LC3B and p62, indicating autophagy impairment which was accompanied by a significant decrease in the expression of collagen.

In addition, siRNA against early autophagic factor *ATG7* (si*ATG7*) were also utilized to inhibit autophagy. *ATG7* expression was knocked down with si*ATG7*, compared to non-targeting control siRNA (Fig. 5D, right panel, lane 3 versus lane 2). Treatment with si*ATG7* caused a significant decrease in LC3B and an increase in p62, indicating inhibited autophagy. Consistently, ductular cells from AAF/CCL4 livers showed a marked increase in the mesenchymal markers, including collagen, vimentin and α-SMA compared to those from normal livers (Fig. 5D, left panel, lane 2 versus lane 1). Knockdown of ATG7 caused diminished expression of mesenchymal markers (Fig. 5D, left panel, lane 3 versus lane 2), indicating that autophagy can contribute to the mesenchymal phenotype of ductular cells.

### Activation of TGF-β/Smad2/3 signaling is required for the regulation of mesenchymal transition by autophagy

**Figure 6:**
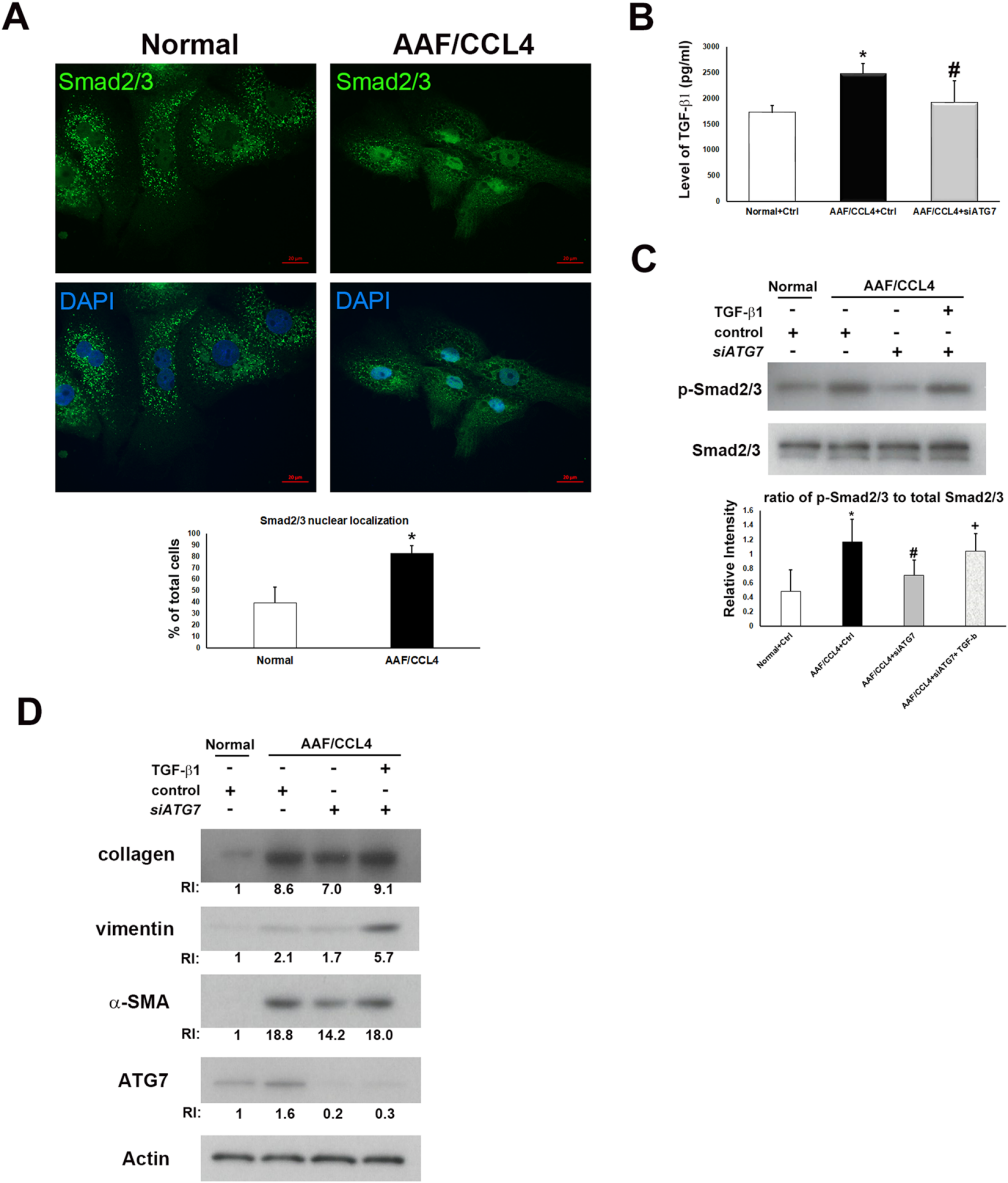
Autophagy-induced TGF-β/Smad2/3 signaling drives mesenchymal transition of ductular cells from AAF/CCL4 livers. (**A**) Immunofluorescence images of Smad2/3 in ductular cells from normal and AAF/CCL4 livers. Nuclear localization was confirmed with DAPI staining. The percentage of Smad2/3 localized in the nucleus was determined by counting 50 immunofluorescence‐positive cells for each sample. (**B**) Cell culture supernatant of ductular cells transfected with control (Ctrl) or siRNA against ATG7 (si*ATG7)* was subjected to ELISA detection for TGF-β1 levels. Data are expressed as mean ± SD from three independent experiments. (**C**) Representative Western blot analysis of phosphorylated (p)- and total Smad2/3 in ductular cells transfected with control or si*ATG7*. The graph below shows the relative intensities of the p-Smad2/3 bands normalized to those for total Smad2/3 from three independent experiments (mean ± SD, n = 3). Full-length blots are presented in Supplementary Fig. S9. Statistical analysis was analyzed by Student’s unpaired t-test. *P < 0.05 compared with normal group; ^#^*P* < 0.05 compared with AAF/CCL4 groups; ^+^*P* < 0.05 compared with AAF/CCL4 with si*ATG7* group. (**D**) Immunoblots and quantification depicting expression of type I collagen, vimentin, α-SMA and ATG7 in ductular cells, which transfected with control or si*ATG7* for 72 hr, and subsequently treated with or without 20 ng/ml TGF-β1 for another 24 hr. Relative intensity (RI) shown was calculated by normalization of the intensities of each marker to the β-actin and to the value of normal cell. Full-length blots are presented in Supplementary Fig. S10.

TGF-β/Smad2/3 signaling has been reported to regulate EMT of ductular cells^9^. We investigated the role of TGF-β/Smad2/3 in regulating autophagy-mediated mesenchymal transition of ductular cells. Immunofluorescence revealed increased nuclear translocation of Smad2/3, which is a hallmark of the activation of TGF-β pathway, in ductular cells from AAF/CCL4 livers (Fig. 6A). Additionally, TGF-β secretion (Fig. 6B) and Smad2/3 phosphorylation (Fig. 6C) were significantly increased in AAF/CCL4 cells compared to normal cells. Inhibition of autophagy by si*ATG7* reduced the production of TGF-β (Fig. 6B) and the ratio of phospho-Smad2/3 to total Smad2/3 (Fig. 6C), demonstrating activation of TGF-β/Smad2/3 signaling in ductular cells from AAF/CCL4 livers in an autophagy-dependent manner.

We further investigated whether TGF-β rescued the mesenchymal phenotype in autophagy-deficient ductular cells. As depicted in Fig. 6D, AAF/CCL4 cells transfected with si*ATG7* exhibited changes in the expression of mesenchymal markers compared with control siRNA (lane 3 verse lane 2). Treatment of recombinant TGF-β (20 ng/ml) substantially upregulated the levels of collagen, vimentin and α-SMA in AAF/CCL4 cells with si*ATG7* compared to untreated cells (lane 4 verse lane 3). These results indicate that autophagy-induced TGF-β/Smad2/3 signaling plays a role in mesenchymal phenotype of ductular cells isolated from AAF/CCL4 livers.

### Co-expression of autophagy and mesenchymal markers in ductular reaction of human cirrhotic livers

**Figure 7:**
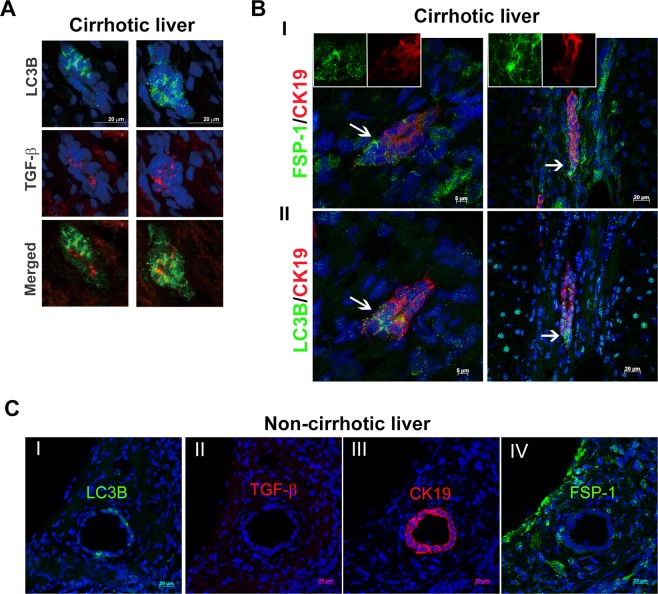
Co-expression of autophagy and mesenchymal markers in the ductular reaction of human cirrhotic livers. A representative series of immunofluorescence results from cirrhotic liver tissues showing expression of LC3B, TGF‐β, FSP-1 and CK19. (**A**) Immunostaining for LC3B and TGF‐β. Merged images show co-expression of LC3B (green) and TGF‐β (red) in ductule structures. (**B**) Panels I and II are consecutive sections from the same cirrhotic tissues. The images in panel I show co-expression of the mesenchymal marker FSP-1 (green) and CK19 (red), and those in panel II show co-expression of LC3B (green) and CK19 (red). The insets in panel I show ductular cells with low expression of CK19 (red) and high expression of FSP-1 (green) that appear to be acquiring a fibroblast morphology. Arrows indicate LC3B-positive ductular cells potentially undergoing the mesenchymal transition during cirrhosis. (**C**) Consecutive sections are from non‐cirrhotic tissue with a bile duct showing regular morphology. The bile duct expressed LC3B (green, panel I) and CK19 (red, panel III) but not TGF‐β (panel II) or FSP-1 (panel IV).

To validate that ductular cells in our system underwent phenotypic conversion into myofibroblasts *in situ*, we examined expression of TGF- β and FSP-1, which is a known marker of the fibroblastic transformation of epithelial cells, and LC3B in human cirrhotic livers. As shown in Fig. 7A and Supplementary Fig. S2, there was an increase of TGF-β in ductular cells demonstrating increases in LC3B. Moreover, the fluorescence of LC3B and FSP-1 was found overlapped in CK19-labeled ductular cells (Fig. 7B and Supplementary Fig. S3, arrows). In FSP-1-expressing cells, a shift toward less cuboidal morphology and a low CK19 expression were observed, indicating that these cells were engaged in a phenotypic transition (Fig. 7B, insets). These findings were also validated in noncirrhotic controls. Unlike those from cirrhotic livers, bile ducts in sections from noncirrhotic livers were negative for both TGF-β and FSP-1 but positive for LC3B and CK19 (Fig. 7C). Collectively, these data show that expression of autophagy markers coincides with expression of TGF-β and the mesenchymal transition of bile ductular cells in cirrhotic livers.

## Discussion

The liver is composed of both parenchymal and nonparenchymal cells with very different phenotypes and functions, and pathological situations may differentially modulate the autophagy status of each cell type^21^. It is believed that autophagy in different types of liver cells may lead to opposite effects on the pathogenesis of fibrosis^11–14,22^. The present study provides evidence that autophagy is increased in the DR of cirrhotic human livers and in ductular cells from AAF/CCL4 rat livers, suggesting the potential to use inhibitors of autophagy as a novel treatment target in liver fibrosis. In contrast to ductular cells, however, cirrhotic human livers showed defective hepatic autophagy, as evidenced by increased accumulation of both LC3B and p62  (Fig. 1B). This observation is consistent with the findings of Ni *et al*., which indicates that loss of autophagy in hepatocytes causes cell death that results in liver fibrosis^22^. Thus, nonspecific anti-autophagy therapies may result in many undesired effects. The cell-specific modulation of autophagy is a prerequisite for developing antifibrotic therapies.

Whether liver epithelial cells could contribute to the development of hepatic scarring through EMT remains controversial^23,24^. Some evidence supports the concept that EMT occurs during various types of human liver injury^7–9^, however, there is a lack of data supporting EMT using *in vivo* lineage tracing experiments^25^. It is possible that EMT in human liver cirrhosis is a state that is not well modeled in rodents in which an extensive DR is not normally observed. Considering the fact that EMT has been extensively studied using *in vitro* liver cell culture model^25^, we have utilized primary culture of ductular cells, which have been isolated from AAF/CCL4-induced cirrhotic livers, to explore the role of autophagy during mesenchymal transition. Our results clearly revealed that ductular cells from AAF/CCL4 livers display morphological and functional characteristics of mesenchymal cells, supporting the notion that ductular cells can undergo trans-differentiation into fibroblasts in progressive liver fibrosis^20^. Furthermore, we show that the mesenchymal transition in DR requires the activation of TGF-β signaling in an autophagy-dependent manner, therefore providing new insights into the connections among autophagy, DR and fibrosis.

DR in humans is an intriguing pathologic feature associated with fibrosis and serves as a source of facultative hepatic progenitor cells (HPCs)^26,27^. HPCs have been reported to exhibit an intermediate epithelial-mesenchymal phenotype^28^. These cells lose mesenchymal markers and gradually attain the complete epithelial phenotype of hepatocytes or cholangiocytes during differentiation. However, this regenerative role of DR/HPCs is still a matter of debate due to its association with poor disease outcome^29–31^. Previous studies from our group have shown that LC3B is increased simultaneously with DR expansion in human cirrhotic livers^15^. This observation, together with the present finding of the involvement of autophagy in controlling the mesenchymal feature of DR cells, raises the possibility that increased autophagy maintains immature DR/HPCs in a proliferative state, ultimately leading to uncontrolled expansion and a failure of differentiation into hepatocytes. Support for this hypothesis comes from the recent observation in which p62 accumulation upon inhibition of autophagy promotes the hepatic differentiation of HPCs^32^. Our unpublished data also indicates that ductular cells under Baf treatment showed increased expression of albumin, a mature hepatocyte marker, suggesting that autophagy inhibitors have therapeutic potential in both reducing fibrosis and supporting parenchymal regeneration.

In conclusion, this study was the first to link autophagy with mesenchymal features of ductular cells in the pathogenesis of cirrhosis. Further studies are needed to fully decipher other potentially involved mechanisms linking autophagy to DR and cirrhosis.

## Materials and Methods

### Human specimens

Liver tissues were obtained from patients at the time of curative resection for hepatocellular carcinoma and were classified into cirrhotic and noncirrhotic groups, according to the pathological examination of the nontumorous liver tissues. The study protocol was approved by the ethics committee of National Taiwan University Hospital (Taipei, Taiwan; no. 201212069RIND). All methods were performed in accordance with the relevant guidelines and regulations. Informed consent was obtained from the subjects.

### Immunohistochemistry

Paraffin sections were deparaffinized and rehydrated. After antigen retrieval, the sections were blocked with 3% hydrogen peroxide and then incubated with antibodies against LC3B (1:500; Sigma) or p62 (1:200; Sigma) at 4 °C overnight. Normal rabbit IgG was used instead of primary antibodies as negative control. Staining was performed using the Envision Dual Link HRP Detection System (Dako) and revealed by 3,3-diaminobenzidine (DAB) chromogen. After counterstaining with hematoxylin, the sections were analyzed under an Axioskop 2 microscope (Carl Zeiss).

### Ductular cell isolation

The rat model of AAF/CCL4-induced liver cirrhosis was established as previously described^15^. Regarding the handling of the rats were performed in accordance with the guidelines established by the Institute of Animal Care and Use Committee of the E-DA Hospital (Kaohsiung, Taiwan). The protocol was reviewed and approved by Institute of Animal Care and Use Committee of the E-DA Hospital (no. EDAH-108003).

Isolation of primary rat ductular cells were performed as previously reported^18^. In brief, the whole livers were dissected, finely diced, and incubated with collagenase type 1 A (2 mg/ml, Sigma) at 37 °C for 30 minutes. The digested liver passed through a 70-μm mesh, and the undigested clot on the mesh was recovered and redigested for an additional 30–60 minutes. The combined filtrate was washed three times in PBS, overlaid on a 33%/77% discontinuous Percoll gradient and then centrifuged at 800 × *g* for 30 minutes. Nonspecific antibody binding was blocked by an antibody against CD32, the FcγΙΙ receptor (BD Biosciences). The cell suspension was incubated with phycoerythrin (PE)-conjugated anti-EpCAM antibody (Santa Cruz Biotechnology) and then anti-PE magnetic particles (BD Biosciences). EpCAM-positive cells were isolated using a separation magnet (BD IMagnet, BD Biosciences) according to the manufacturer’s instructions. The supernatant is the negative fraction. Expression of EpCAM in the negative and positive fractions was analyzed using a FACSVerse flow cytometer (BD Biosciences).

### Culture and treatment of primary rat ductular cells

The isolated immunopositive faction was suspended in plating medium composed of DMEM/F12 supplemented with 10% FBS, 1X insulin-transferrin-selenium (ITS, Gibco), EGF (10 ng/ml), cholera toxin (10 ng/ml) and hydrocortisone (0.4 μg/ml). After allowing the cells to attach for 48 hours, the plating medium was replaced with growth medium containing a reduced concentration of serum (5% FBS) and HGF (10 ng/ml). Cells were grown in growth medium for 24 hours and then treated with bafilomycin A1 (100 nM) for an additional 24 hours.

### Transfection

Primary rat ductular cells were transfected with 100 nM ATG7-targeted siRNA (SMARTpool Rat ATG7, L095596-01) using DharmaFECT 1 siRNA transfection reagent (GE Dharmacon) following the manufacturer’s instruction. Transfections were carried out in 12-well plates and incubated cells for 72 hours for protein analysis. The efficiency of gene silencing was assessed by Western blot analysis as described below.

### Western blot analysis

Total protein extracts were prepared as previously described^33^. The membranes were probed with the following antibodies: rabbit anti-LC3B (1:4000; Sigma), mouse anti-ATG5 (1:1000; Sigma), rabbit anti-ATG7 (1:4000; Sigma), rabbit anti-p62/SQSTM1 (1:4000; Sigma), rabbit anti-collagen (1:1000; Abcam), mouse anti-SMA (1:4000; Sigma), mouse anti-vimentin (1:4000; Sigma), mouse anti-β-actin (1:10000; Novus), rabbit anti-phospho-Smad2/3 (1:1000; Cell Signaling) and rabbit anti-Smad2/3 (1:1000; Cell Signaling). After an overnight incubation with each primary antibody, the membranes were incubated with horseradish peroxidase-conjugated secondary antibody. The proteins were then detected using an enhanced chemiluminescence detection system (Thermo). Densitometric analysis was performed using Image J software (NIH). Relative intensities were calculated by normalizing the intensities of each marker to the loading control.

### Dual immunofluorescence staining

Cell cultures grown for 3–4 days were fixed with 4% paraformaldehyde, permeabilized with 0.5% (v/v) Triton X-100 and blocked with 4% bovine serum albumin solution in PBS at room temperature. Cells were incubated overnight at 4 °C with primary antibodies, washed, incubated with Alexa Fluor 488- and Alexa Fluor 594-conjugated specific secondary antibodies (Invitrogen), mounted with Vectashield-DAPI (Vector Laboratories), and observed under a fluorescence microscope (Axio Imager M1, Carl Zeiss) or a confocal microscope (LSM780, Carl Zeiss).

### Transmission electron microscopy

The sample was prefixed in 2.5% glutaraldehyde for 1.5 hours, washed twice with 0.1 M PBS (pH 7.0), and postfixed in 2% osmium tetraoxide for 1 hour. Dehydration was then performed in an ascending ethanol gradient. Samples were polymerized using Spurr resin at 72 °C for 12 hours. Ultrathin sections were cut with a Leica UC7 ultramicrotome and ultrastructural examination was performed on a Hitachi HT7700 transmission electron microscope with 100 kV acceleration voltage.

### Quantitative real-time polymerase chain reaction (PCR)

Total RNA was extracted from ductular cells using Trizol reagent (Invitrogen). Two microgram total RNA was reverse transcribed into cDNA using a first-strand cDNA synthesis kit (Clontech). Quantitative real-time PCR was performed with SYBR Green Master Mix (Bio-Rad) on the CFX Connect real-time PCR detection system (Bio-Rad). Primers used include: LC3B forward primer, 5′-CAGGTTGCCTAGCAGAGGTC-3′; LC3B reverse primer, 5′-TGTCCTATACACCTGACCTGTTTC-3′; p62 forward primer, 5′-GCCCTGTACCCACATCTCC-3′; p62 reverse primer, 5′-CCATGGACAGCATCTGAGAG-3′; β-actin forward primer, 5′-GGAGATTACTGCCCTGGCTCCTA-3′; β-actin reverse primer, 5′-GACTCATCGTACTCCTGCTTGCTG-3′. Relative levels of mRNA expression were calculated using the ΔΔCT method using β-actin levels for normalization.

### Transwell migration assay

2 × 10^4^ cells were plated onto the upper chamber of the 24-well transwell plate in serum-free DMEM/F12. The lower chamber contained DMEM/F12 supplemented with 10% FBS. The cells were allowed to migrate through the membrane towards the lower chamber for 18 hours at 37 °C. The non-migrated cells on the upper chamber were removed using a cotton swab, and the cells present underneath the membrane were fixed with cold ethanol for 10 minutes and stained with 0.2% crystal violet. For quantification, migrated cells were lysed with 10% acetic acid and counted in a spectrophotometer at OD590 nm.

### Statistical analysis

All results are expressed as mean ± SD of at least three independent experiments. Statistical analysis was performed using a two-tailed unpaired Student’s t test. Differences were regarded statistically significant with *P* < 0.05.

## Supplementary information

supplementary information

## Data Availability

The datasets generated and analyzed during the current study are available from the corresponding author upon reasonable request.
