# Supplementary material for: A critical role of autophagy in regulating the mesenchymal transition of ductular cells in liver cirrhosis
Source: Sci Rep. 2019 Jul 23;9:10673. doi: 10.1038/s41598-019-46764-x (PMC6650611; doi:10.1038/s41598-019-46764-x)
Supplement: Supplementary file 1 — supplementary information [file 41598_2019_46764_MOESM1_ESM.docx]

**A critical role of autophagy in regulating the mesenchymal transition of ductular cells in liver cirrhosis**

Tzu-Min Hung^1,2^*, Yu-Jen Huang^1^*, Yu-Chun Lin^2^, Yu-Hsuan Chen^4^, Yao-Ming Wu^1#^, Po-Huang Lee^1,3#^

^1^Department of Surgery, National Taiwan University Hospital and National Taiwan University College of Medicine, Taipei, Taiwan;

^2^Department of Medical Research, E-DA Hospital, Kaohsiung, Taiwan

^3^Department of Surgery, E-DA Hospital, Kaohsiung, Taiwan

^4^Department of Internal Medicine, National Taiwan University Hospital and National Taiwan University College of Medicine, Taipei, Taiwan

*These authors contributed equally to this work.

# Correspondence: pohuang1115@ntu.edu.tw; wyaoming@gmail.com


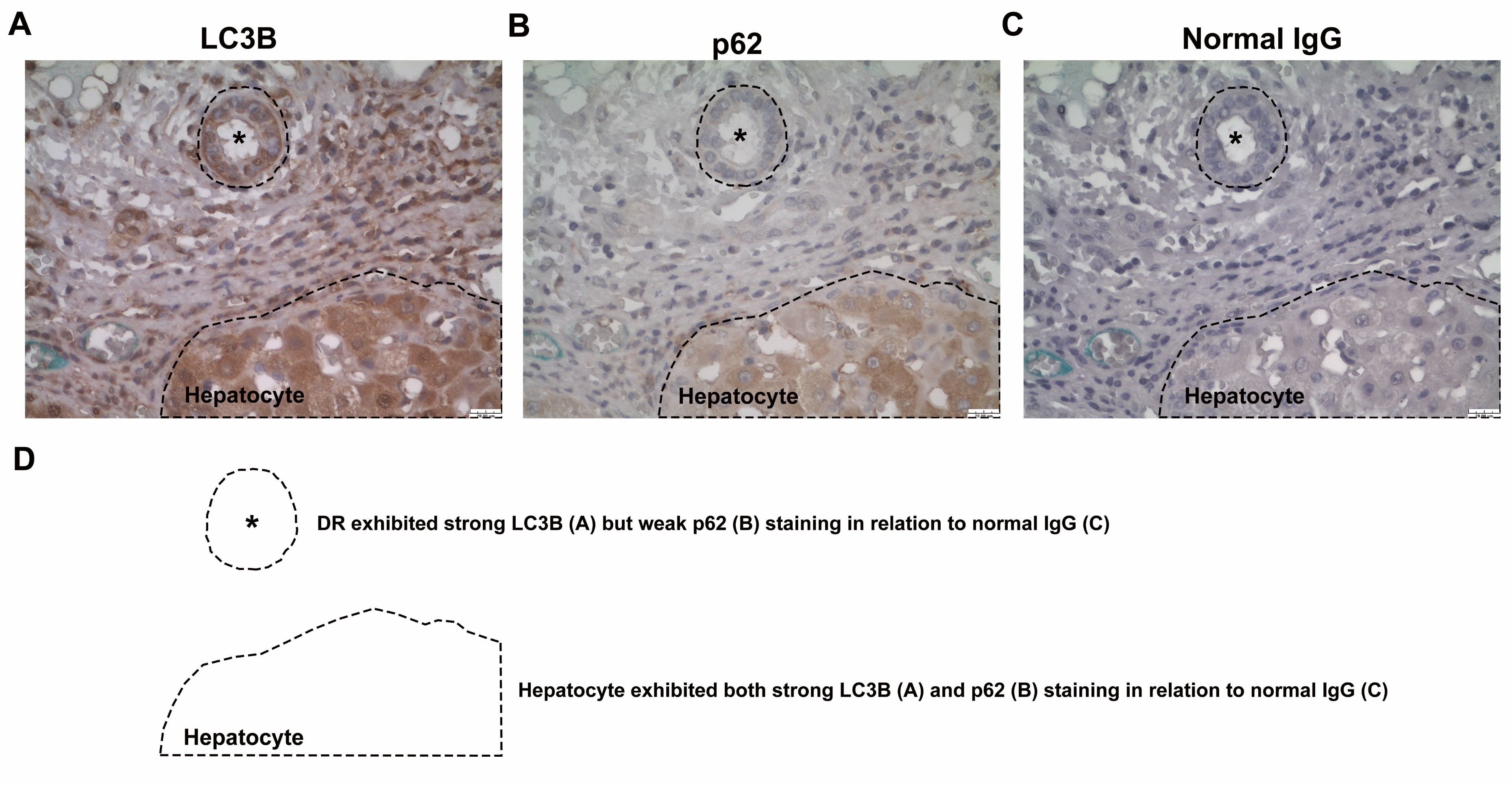


**Supplementary Fig. 1:** Consecutive sections are from a cirrhotic liver tissue containing both portal area and periportal parenchymal region. Immunohistochemical detection of autophagy markers LC3B (**A**) and p62 (**B**). Normal IgG is used for negative control counterstain (**C**). Scale bar, 20 μm. (**D**) Depicting of the staining results. Ductular reaction (DR), which is marked by asterisk, localized in portal area and showed expression of LC3B but not of p62. Hepatocyte, the main parenchymal cell, localized in periportal parenchyma region and showed expression of both LC3B and p62.


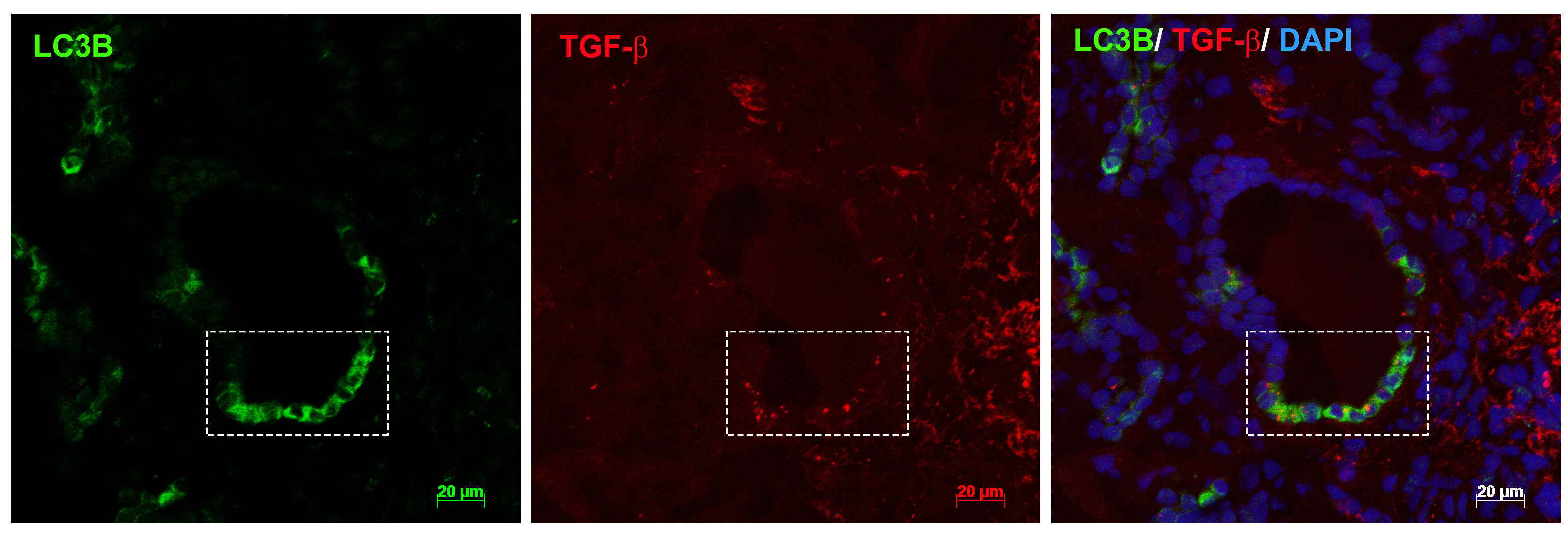


**Supplementary Fig. 2:** Immunofluorescence staining for LC3B and TGF-β in a patient with cirrhosis. Merged images show co-expression of LC3B (green) and TGF‐β (red) in ductule structures. The dotted rectangles show an increase of TGF-β in ductular cells demonstrating increases in LC3B.


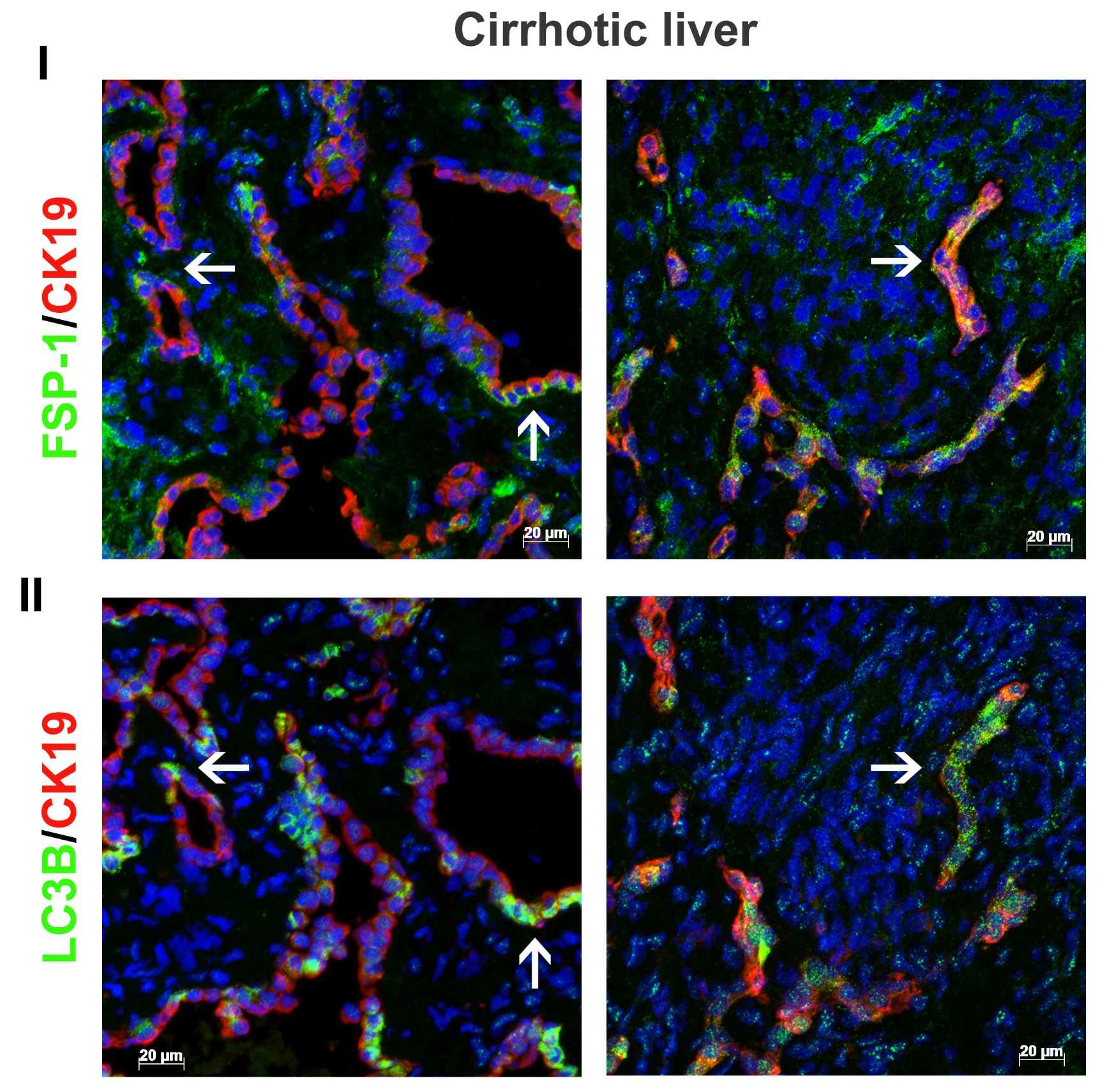


**Supplementary Fig. 3: Co-expression of autophagy and mesenchymal markers in the ductular reaction of human cirrhotic livers.** Panels I and II are consecutive sections from the same cirrhotic tissues. The images in panel I show coexpression of the mesenchymal marker FSP-1 (green) and CK19 (red), and those in panel II show coexpression of LC3B (green) and CK19 (red). Arrows indicate the fluorescence of LC3B and FSP-1 was overlapped in CK19-labeled ductular cells.

­­­
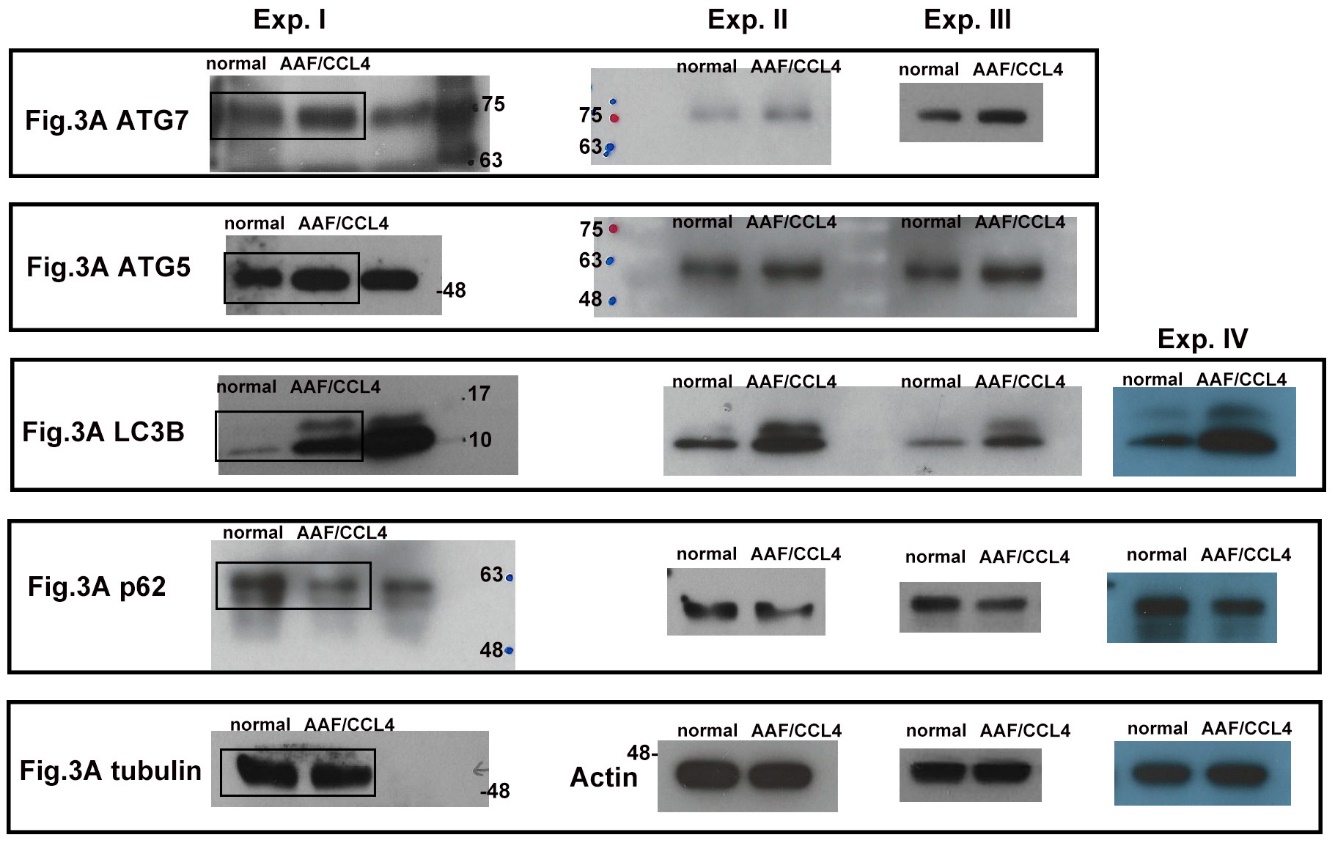


**Supplementary Fig. 4:** Full-length pictures of the blots related to Figure 3A. The raw blotting data of 3-4 experiments are presented. Results shown in Figure 3A are cropped from the experiment III (Exp. III). Molecular weight (in kDa) are indicated.


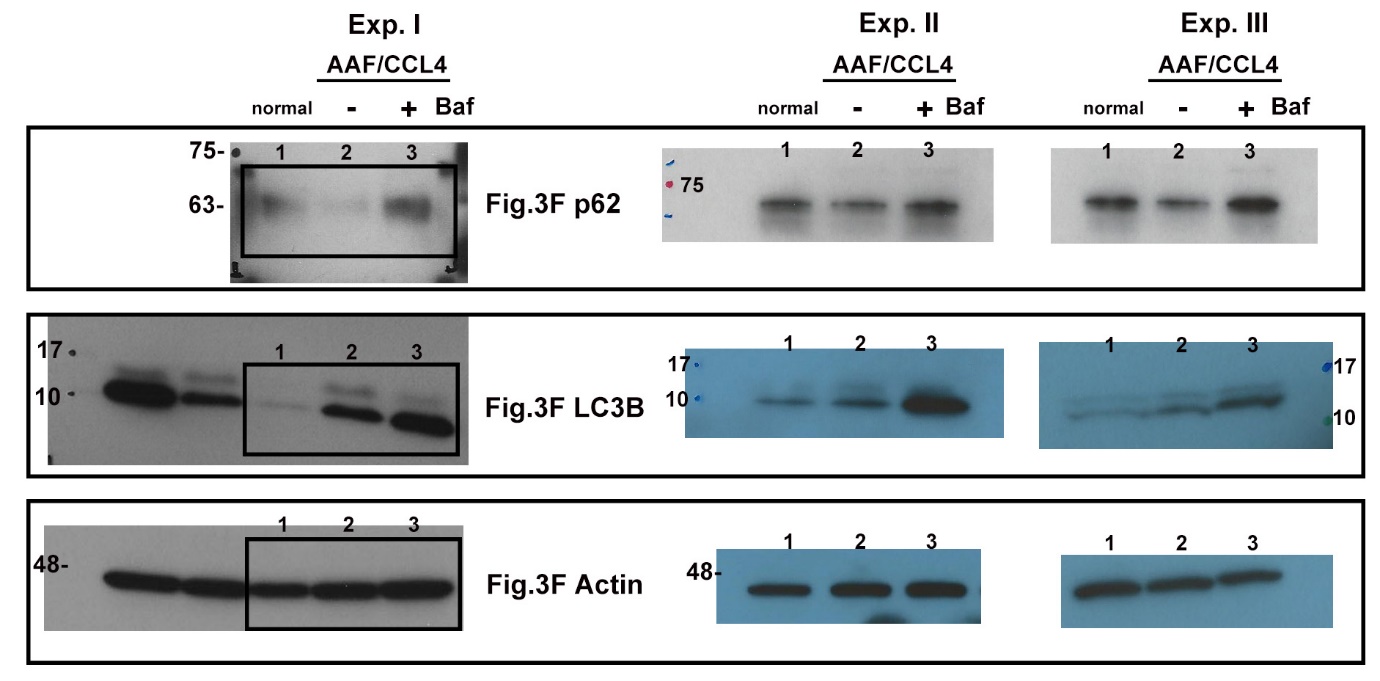


**Supplementary Fig. 5:** Full-length pictures of the blots related to Figure 3F. The raw blotting data of 3 experiments are presented. Results shown in Figure 3F are cropped from the experiment II (Exp. II). Molecular weight (in kDa) are indicated.


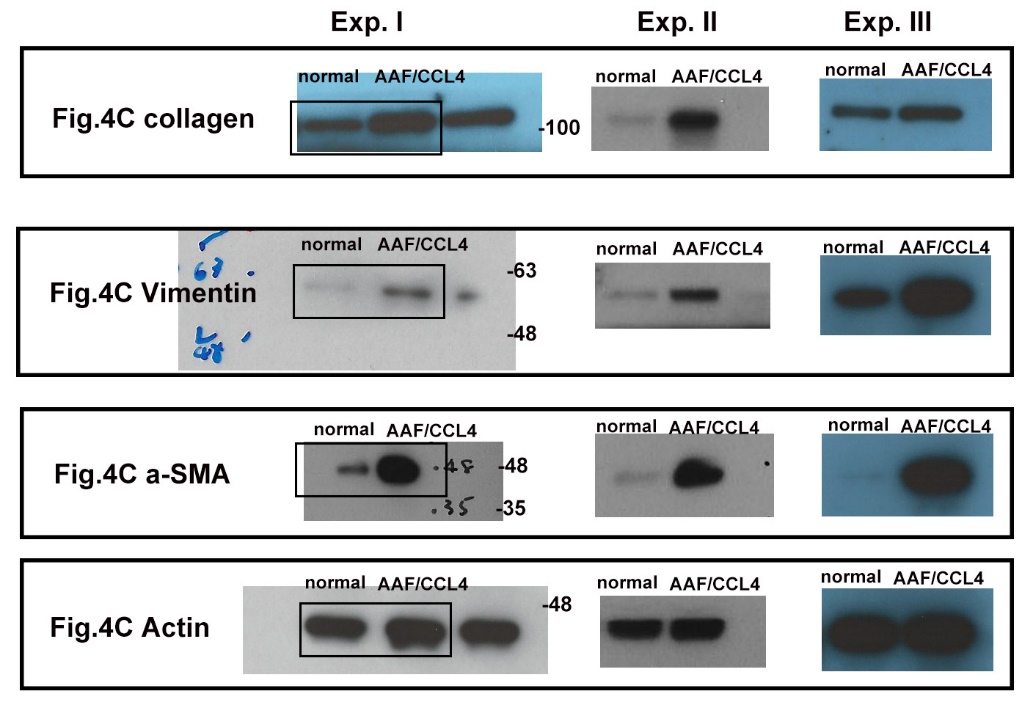


**Supplementary Fig. 6:** Full-length pictures of the blots related to Figure 4C. The raw blotting data of 3 experiments are presented. Results shown in Figure 4C are cropped from the experiment II (Exp. II). Molecular weight (in kDa) are indicated.


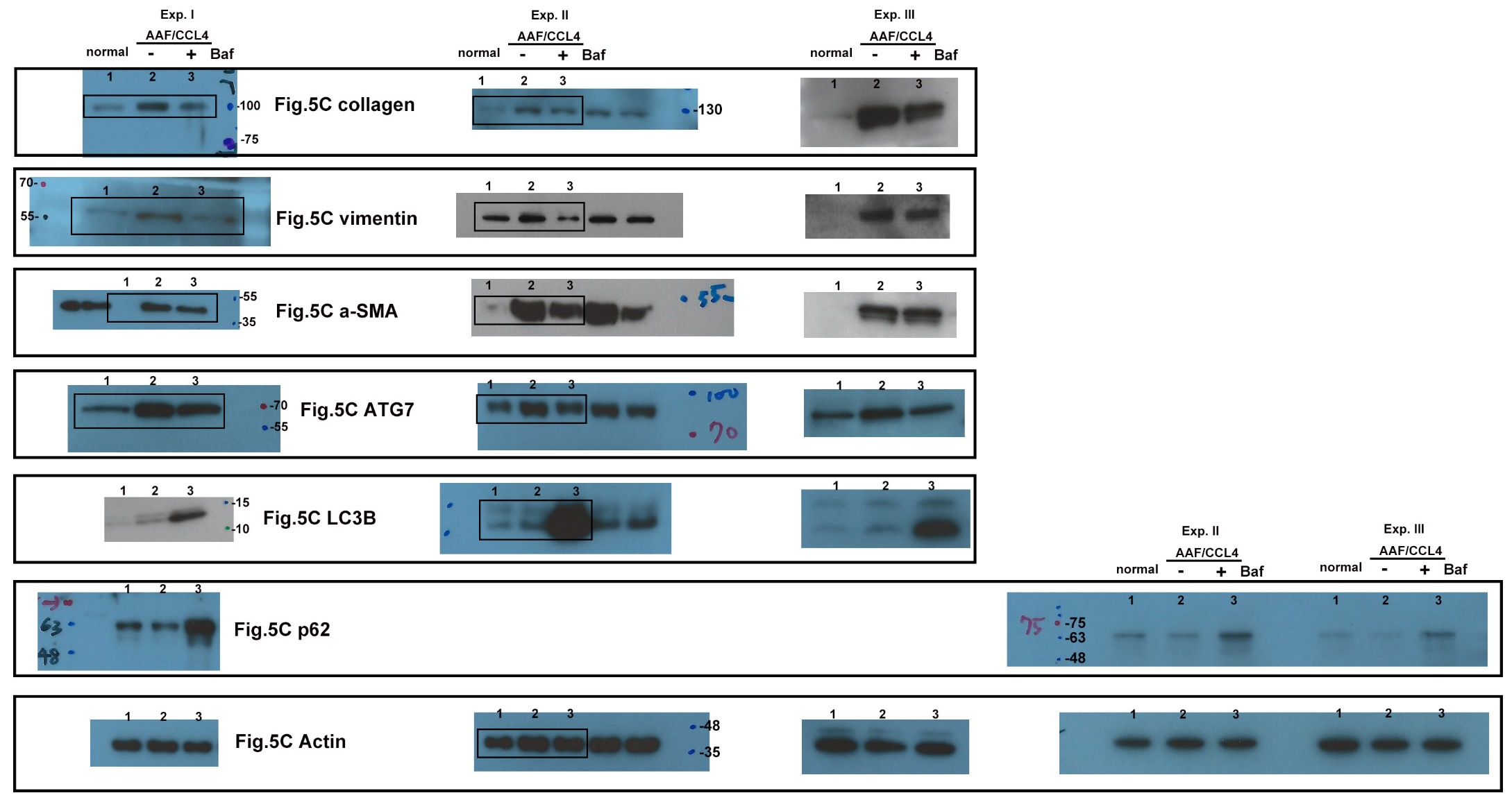


**Supplementary Fig. 7:** Full-length pictures of the blots related to Figure 5C. The raw blotting data of 3~4 experiments are presented. Results of mesenchymal markers shown in Figure 5C are cropped from the experiment II (Exp. II). Results of autophagic markers shown in Figure 5C are cropped from the experiment III (Exp. III) Molecular weight (in kDa) are indicated.


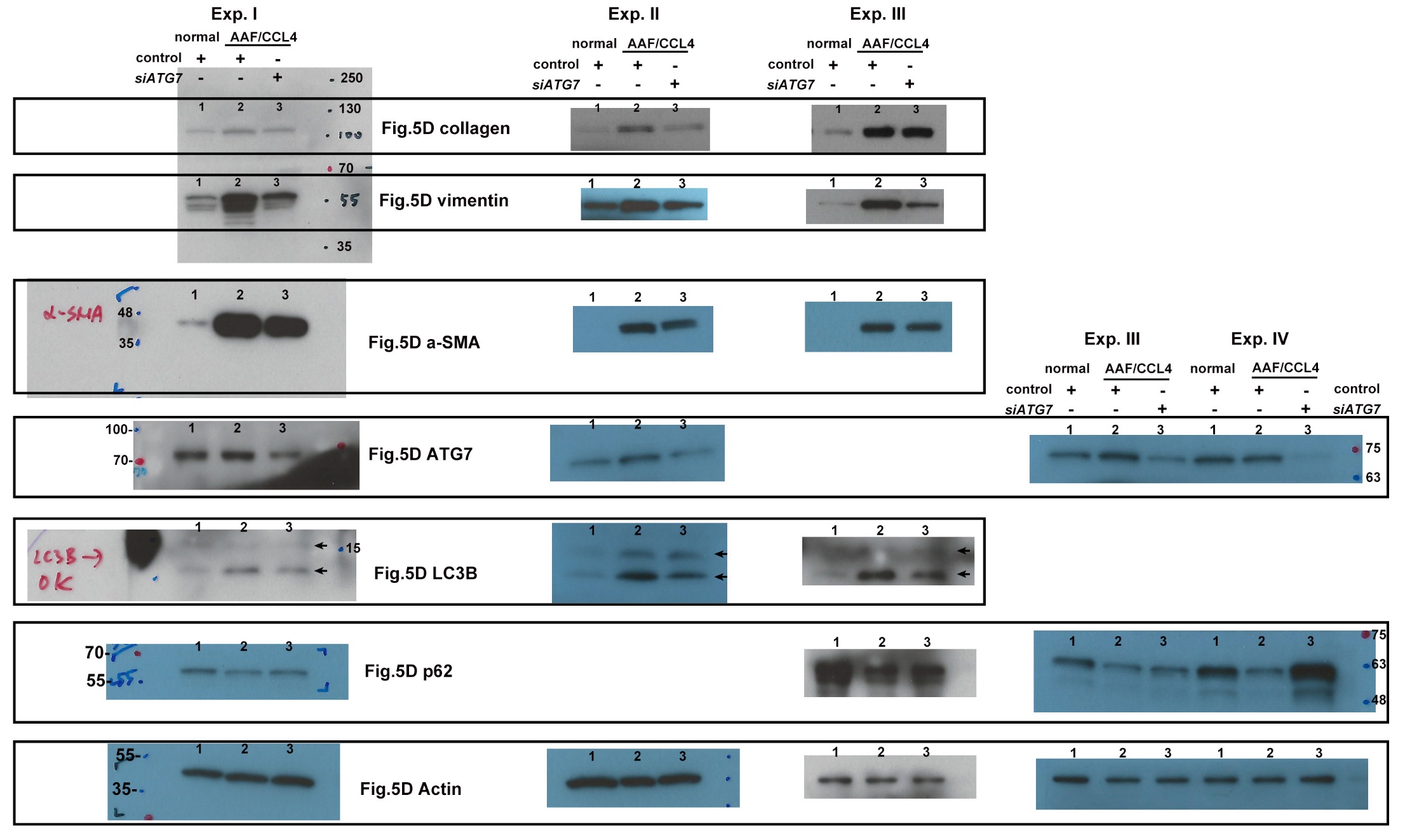


**Supplementary Fig. 8:** Full-length pictures of the blots related to Figure 5D. The raw blotting data of 3~4 experiments are presented. Results of mesenchymal markers shown in Figure 5D are cropped from the experiment I (Exp. I). Results of autophagic markers shown in Figure 5C are cropped from the experiment IV (Exp. IV). Molecular weight (in kDa) are indicated.


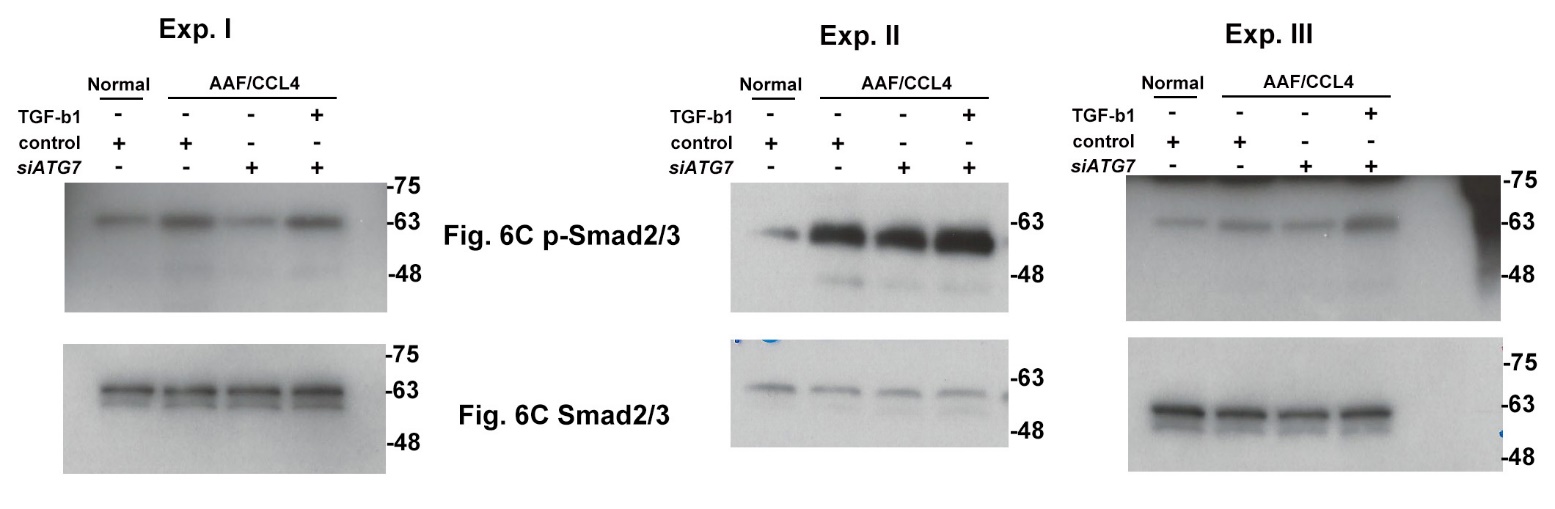


**Supplementary Fig. 9:** Full-length pictures of the blots related to Figure 6C. The raw blotting data of 3 experiments are presented. Results shown in Figure 6C are cropped from the experiment I (Exp. I). Molecular weight (in kDa) are indicated.


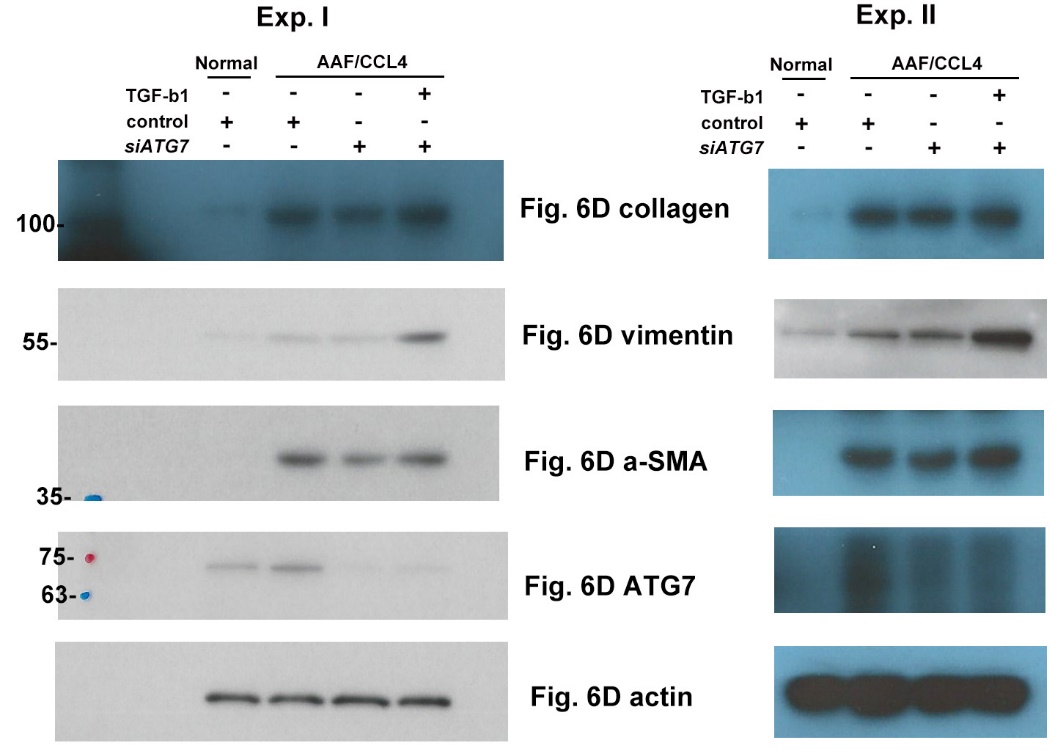


**Supplementary Fig. 10:** Full-length pictures of the blots related to Figure 6D. The raw blotting data of 2 experiments are presented. Results shown in Figure 6D are cropped from the experiment I (Exp. I). Molecular weight (in kDa) are indicated.
